# Ovarian Responses and Outcomes of In Vitro Fertilization Following Progesterone-Primed Ovarian Stimulation and Gonadotropin-Releasing Hormone (GnRH) Antagonist Protocols Using Follitropin Delta

**DOI:** 10.7759/cureus.85341

**Published:** 2025-06-04

**Authors:** Masachi Hanaoka, Kanako Hanaoka, Mayu Yamada

**Affiliations:** 1 Department of Reproductive Medicine, Hanaoka In Vitro Fertilization (IVF) Clinic Shinagawa, Tokyo, JPN

**Keywords:** controlled ovarian stimulation (cos), dydrogesterone, follitropin delta, gnrh antagonist protocol, progesterone-primed ovarian stimulation (ppos) protocols

## Abstract

Aim: This study aimed to examine the effects on oocyte retrieval, culture results, and pregnancy rates in Japanese women who underwent ovarian stimulation for assisted reproductive technology using two different protocols with follitropin delta. Specifically, it compared ovarian responses between a gonadotropin-releasing hormone (GnRH) antagonist protocol, used as a conventional controlled ovarian stimulation method, and a progesterone-primed ovarian stimulation (PPOS) protocol, considered a relatively new method.

Materials and methods: This retrospective, observational study was conducted at a single in vitro fertilization clinic in Tokyo, Japan, from April 2022 to March 2024. The study population included infertile patients who were scheduled for treatment and met the inclusion criteria. Eligible participants were Japanese women aged 20-45 years with preserved ovarian function, indicated by an anti-Müllerian hormone level of ≥0.8 ng/mL. Exclusion criteria included contraindications for follitropin delta, previous oocyte stimulation for egg donation or fertility preservation, follicle-stimulating hormone levels ≥35 mIU/mL, uncontrolled malignant disease, stage III/IV endometriosis, and the use of hormone preparations (excluding thyroid medication) during the last menstrual cycle prior to study entry. The primary endpoint was the pregnancy rate per transfer in GnRH antagonist and PPOS cycles. Secondary endpoints included the number of oocytes retrieved and the blastocyst formation rate. Ongoing pregnancy was defined as a pregnancy in which a fetal heart rate was confirmed by 10 weeks of gestation. Accumulated data from GnRH antagonist cycles and PPOS cycles at the institution were combined to compare oocyte retrieval outcomes and pregnancy rates per transfer. Additionally, a stratified analysis by age (<35, 35-39, and ≥40 years) was performed.

Results: Accumulated data included 149 GnRH antagonist cycles and 147 PPOS cycles. Oocyte retrieval outcomes and pregnancy rates per transfer were compared between the two protocols. There was no significant difference in the number of oocytes retrieved. However, the number of blastocysts showed a higher trend in the PPOS group compared to the GnRH antagonist group (p = 0.065). Across all age groups, the PPOS cycle tended to yield higher numbers of retrieved oocytes and blastocysts. Nonetheless, no significant difference was observed in the pregnancy rate per transfer between the two protocols.

Conclusions: Both the GnRH antagonist and PPOS protocols demonstrated trends toward higher numbers of retrieved oocytes and blastocysts, with comparable pregnancy rates across all age groups, suggesting similar clinical outcomes. A key limitation of this study is its retrospective design at a single institution; therefore, future prospective, large-scale studies are warranted.

## Introduction

In performing controlled ovarian stimulation (COS) based on assisted reproductive technologies (ART), GnRH analogs, i.e., gonadotropin-releasing hormone (GnRH) agonists and GnRH antagonists, are used to regulate pituitary function and prevent premature luteinization by suppressing the production of endogenous follicle-stimulating hormone (FSH) and luteinizing hormone (LH). A premature LH surge, caused by multiple growing follicles producing increased plasma estradiol, is a significant drawback of COS [1]. GnRH agonists were commonly used in the past; however, in recent years, GnRH antagonists have become the standard of care in COS treatment due to several advantages: greater patient friendliness, shorter duration of administration, improved convenience [2], and enhanced safety through the reduction or elimination of ovarian hyperstimulation syndrome (OHSS) via GnRH agonist triggering and a freeze-all policy [3]. Nonetheless, despite these advantages, GnRH antagonists are still administered via injection and are associated with high therapy costs.

With the increasing use of freeze-all protocols, numerous studies have explored new regimens for controlling the LH surge in freeze-all cycles. In progesterone-primed ovarian stimulation (PPOS) protocols, the use of oral progestins has been proposed [4], based on physiological evidence that progesterone can block the pre-ovulatory LH surge by directly inhibiting estradiol-responsive cells in the hypothalamus. Progesterone also exerts a rapid but transient disruptive effect on estradiol [5].

The PPOS protocol is a novel form of COS first reported by Kuang et al. [6]. It is viewed as a method that reduces both pain and cost for patients, and its use has been increasing in Japan in recent years [7]. Over the past few years, in vitro fertilization (IVF) treatments have been covered by health insurance in Japan. PPOS, which is easy to manage and reduces the number of hospital visits, has therefore gained popularity. However, no concrete data supporting this trend has yet been made public.

The random start protocol and two-stage oocyte retrieval have gained more widespread use in recent years. It has been found that even when endogenous progesterone is secreted during the luteal phase, COS remains possible, and good-quality, mature oocytes can still be obtained. PPOS is a COS method that suppresses the early LH surge using progestin. It has been widely used in recent years because it yields treatment outcomes comparable to those of the conventional GnRH antagonist protocol, is inexpensive, and can be administered orally.

However, a drawback of PPOS is that it induces early decidualization of the endometrium, which precludes fresh embryo transfer. Therefore, it is predicated on the use of a freeze-all protocol. PPOS protocols have emerged as an effective alternative to GnRH antagonist protocols in treatments where a receptive endometrium is not required, such as freeze-all IVF cycles (e.g., preimplantation genetic screening, oocyte donation, fertility preservation) [8,9]. In recent years, various types of progestins, including medroxyprogesterone, micronized progesterone, desogestrel, and dydrogesterone, have been evaluated in freeze-all IVF cycles as regimens to control the LH surge. The number of oocytes retrieved and the clinical outcomes appear to be similar to those of GnRH antagonist cycles [1,6,10].

When focusing only on PPOS protocols that use follitropin delta (Rekovelle, Ferring Pharmaceuticals Co., Ltd., Tokyo, Japan), there have been few reported comparisons with GnRH antagonist protocols, as nearly all previous studies have focused on follitropin alpha and follitropin beta [11]. Follitropin delta is a recombinant FSH preparation, with daily dosage determined based on the patient’s anti-Müllerian hormone (AMH) level and body weight. It employs an individualized dosing algorithm, with no dose adjustments during the stimulation period. Follitropin delta is the first recombinant FSH preparation manufactured using human-derived cells and is expected to exhibit enhanced physiological function in vivo.

According to the individualized dosing algorithm, the dosage is higher in patients with lower AMH levels or higher body weight and lower in those with higher AMH levels or lower body weight. The daily dosage is calculated to retrieve an optimal number of eight to 14 oocytes. To define individual doses for ovarian stimulation, reduce the risk of OHSS, and improve reproductive outcomes, clinicians and health policymakers have proposed classifying patients based on individual characteristics and/or markers of ovarian reserve [12-14].

In previous studies, including clinical trials, most reports on individualized follitropin delta usage have involved fresh embryo transfer. Reports involving frozen embryo transfer are limited. All currently reported systematic reviews and meta-analyses of follitropin delta focus on cases involving fresh embryo transfer [15-17]. Evidence regarding its use in frozen embryo transfer, which is increasing globally, remains limited [18]. Additionally, nearly all pituitary suppression protocols utilize GnRH agonists or GnRH antagonists, and reports on PPOS protocols using follitropin delta are scarce. Further investigation, particularly in Japanese women, is considered necessary.

Previous studies using other medications have compared the GnRH antagonist protocol with the PPOS protocol; however, there are still limited reports on COS using follitropin delta. Furthermore, no studies have analyzed the data based on AMH levels or age. Therefore, this study aimed to compare ovarian response and pregnancy outcomes between PPOS and GnRH antagonist protocols using follitropin delta. The primary outcome was the clinical pregnancy rate per embryo transfer.

This study investigated cases using follitropin delta and compared the ovarian responses with those of the GnRH antagonist protocol, a conventional COS method, and PPOS, a relatively new COS method. The aim was to evaluate the effects on oocyte retrieval, culture outcomes, and pregnancy rates in Japanese women who underwent ovarian stimulation for ART.

## Materials and methods

This retrospective, observational study was conducted at Hanaoka IVF Clinic Shinagawa, Tokyo, Japan, from April 2022 to March 2024. The study was approved by the Ethics Committee of Hanaoka IVF Clinic Shinagawa and was prospectively registered at Clinical Trials (Osk-07-003). The study population included infertile patients planning to undergo treatment who fulfilled the inclusion criteria and had signed the appropriate informed consent form.

The inclusion criteria were as follows: (1) Japanese women aged 20-45 years with preserved ovarian function and AMH ≥0.8 ng/mL; (2) BMI between 18 and 30 kg/m²; (3) a history of undergoing IVF with up to three oocyte retrievals; (4) oocyte retrieval performed by a skilled physician at our institution; and (5) freeze-all cycles. The exclusion criteria included contraindications to follitropin delta, undergoing oocyte stimulation for egg donation or fertility preservation, ovarian dysfunction with FSH ≥35 mIU/mL or a diagnosis of primary ovarian insufficiency, uncontrolled malignant disease, stage III/IV endometriosis, and the use of hormone preparations (excluding thyroid medication) during the last menstrual cycle before study entry.

Stimulation was initiated on the second or third day of the menstrual cycle or, in cases where oral contraception had been used, after at least one cycle drug holiday.

Treatment allocation to the intervention was fixed as COS with a GnRH antagonist protocol (from April 2022 to June 2023) and with PPOS (from July 2023 to March 2024). In the GnRH antagonist cycle, endogenous LH suppression was achieved by subcutaneous injections of cetrorelix (Cetrotide for Injection 0.25 mg, Merck Biopharma Co., Ltd., Tokyo, Japan), starting on day 5 or 6 of COS and continuing until ovulation triggering [13,19]. In the PPOS cycle, the LH surge was controlled by oral administration of 10 mg dydrogesterone (Duphaston, Viatris, Inc., Tokyo, Japan) twice daily from stimulation day 2 until the day of the trigger.

Blood samples and transvaginal ultrasounds were performed before the administration of any medication and on stimulation days 6, 8-10, and the day of the trigger. Final oocyte maturation was induced with a bolus of GnRH agonist (Buserecure 0.5 mL, Fuji Pharma Co., Ltd., Tokyo, Japan) and 5,000 units of urinary HCG (HCG MOCHIDA for Intramuscular Injection 5,000 units, Mochida Pharmaceutical Co., Ltd., Tokyo, Japan) when at least three follicles >18 mm were observed on transvaginal ultrasound in both cycles. Oocyte retrieval was performed approximately 36 hours thereafter.

Fertilization was performed on the same day using either conventional IVF or intracytoplasmic sperm injection. After confirming fertilization the following day, embryos were cultured, and all blastocysts were cryopreserved via vitrification (a freeze-all strategy). Frozen-thawed embryo transfer was carried out in both cycles during a hormone replacement cycle.

Starting from the second or third day of the menstrual cycle, estradiol (Estrana 4 mg, Hisamitsu Pharmaceutical Co., Inc., Saga, Japan) was administered using two patches every other day. Endometrial thickness was checked around day 14 of the menstrual cycle, and if it was ≥8 mm, vaginal administration of a progesterone suppository (Lutinus, Ferring Pharmaceuticals Co., Ltd., Tokyo, Japan, or UTROGESTAN vaginal capsules, Fuji Pharma Co., Ltd., Tokyo, Japan) was initiated at 300 mg/day. Frozen-thawed blastocyst transfer was then performed vaginally 5-6 days after the start of progesterone administration.

There were no cases of OHSS requiring hospitalization in either group. The primary endpoint was the pregnancy rate in GnRH antagonist or progesterone-downregulated cycles. Secondary endpoints included the number of oocytes and the blastocyst formation rate. For the ongoing pregnancy rate, cases were defined as those in which a fetal heart rate could be confirmed up to 10 weeks.

**Figure 1:**
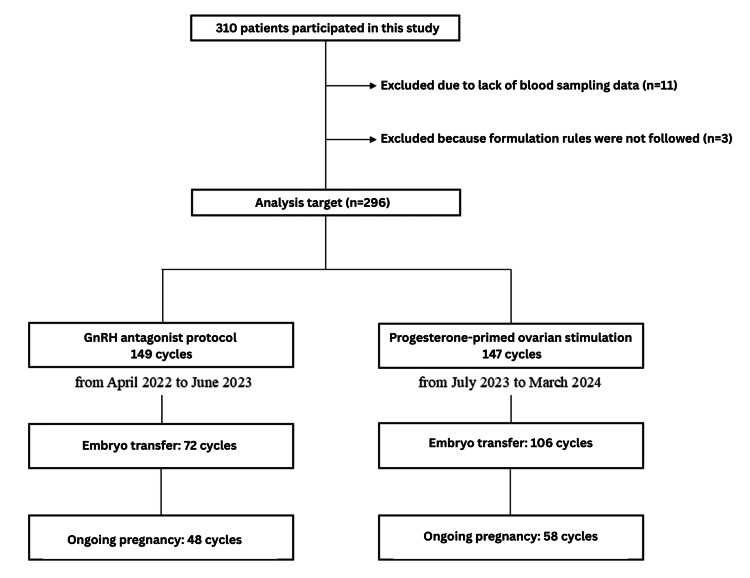
Study flow diagram Treatment allocation to the intervention was fixed to COS with a GnRH antagonist protocol (from April 2022 to June 2023) and with PPOS (from July 2023 to March 2024). GnRH: gonadotropin-releasing hormone, COS: controlled ovarian stimulation, PPOS: progesterone-primed ovarian stimulation

Accumulated data from GnRH antagonist cycles and PPOS cycles at our institution were combined, and the oocyte retrieval results and pregnancy rate per transfer were compared. Additionally, a stratified analysis by age (<35, 35-39, ≥40 years) was conducted. For the accumulated data from GnRH antagonist cycles, this study analyzed a fixed protocol in which the GnRH antagonist was administered on day 5 or 6. The dosage of follitropin delta covered by national health insurance in Japan is a maximum of 12 µg per day. Patients in whom it was effectively administered with off-label use (n=3) were excluded from the analysis. Individuals with missing data for AMH, FSH, LH, or estradiol levels were also excluded (n=11). From the total combined data of 310 cycles, 14 cycles were excluded based on the above criteria, resulting in an analysis of 296 cycles. The study flow is shown in Figure 1.

Statistical analysis was performed using JMP version 18 (SAS Institute Inc., Cary, NC, USA). Univariate analysis was conducted using Student’s t-test or Fisher’s exact test. For multivariate analysis, a multiple regression model adjusted for AMH level was used. When trends in differences for AMH, FSH, and LH levels between cycles were observed, a multivariate logistic regression analysis was performed, including these three factors as moderating variables. Odds ratios and confidence intervals were estimated, and a P value of less than 5% was considered statistically significant.

## Results

The accumulated data for GnRH antagonist cycles (n=149) and PPOS cycles (n=147) at our institution were combined, and the oocyte retrieval outcomes and pregnancy rate per transfer were compared. The study flow diagram is shown in Figure 1. A stratified analysis by age was also conducted. Baseline characteristics are presented in Table 1; the baseline FSH level in the PPOS group was significantly higher, while no significant differences were observed in other variables.

**Table 1:** Baseline characteristics The baseline FSH in the PPOS cycle was significantly higher, but no significant differences were seen in other items. *p<0.05: Student's t-test or Fisher’s exact test. GnRH: gonadotropin-releasing hormone, PPOS: progesterone-primed ovarian stimulation, AMH: anti-Müllerian hormone, FSH: follicle-stimulating hormone, LH: luteinizing hormone, E2: estradiol

Variables	GnRH antagonist cycles (n=149 cycles)	PPOS cycles (n=147 cycles)	p-value
Age (y)	36.4 ± 4.0	36.9 ± 3.8	0.195
<35	48 (32.2%)	38 (25.9%)	0.476
35-39	66 (44.3%)	70 (47.6%)	
≥40	35 (23.5%)	39 (26.5%)	
AMH (ng/mL)	3.05 ± 2.20	2.64 ± 2.20	0.114
<1.2	34 (22.8%)	39 (26.5%)	0.503
1.2-5.0	88 (59.1%)	88 (59.9%)	
≥5.0	27 (18.1%)	20 (13.6%)	
No. of days of ovarian simulation	9.1 ± 1.6	9.4 ± 1.5	0.145
Total Rekovelle dosage (µg)	82.8 ± 23.9	89.0 ± 25.2	0.030*
Daily Rekovelle dosage (µg)	9.2 ± 2.6	9.6 ± 2.5	0.221
Baseline FSH level (mIU/mL)	7.9 ± 2.8	9.1 ± 3.1	<0.001*
Baseline LH level (mIU/mL)	4.7 ± 2.6	4.5 ± 2.3	0.335
E2 level at time of oocyte retrieval (pg/mL)	1995.8 ± 1316.7	2320.4 ± 1655.0	0.063

The number of oocytes retrieved and the number of blastocysts formed in the GnRH antagonist cycle versus the PPOS cycle were 8.9 ± 6.4 versus 9.4 ± 5.6 (mean ± SD) retrieved oocytes and 3.4 ± 3.2 versus 4.1 ± 3.2 blastocysts, respectively. The results of the univariate analysis for the number of retrieved oocytes were 8.9 ± 6.4 versus 9.4 ± 5.6 (p=0.465), and for the number of blastocysts were 3.4 ± 3.2 versus 4.1 ± 3.2 (p=0.065). No significant difference was observed in the number of retrieved oocytes between the two cycles; however, the number of blastocysts showed a trend toward being higher in the PPOS cycle compared to the GnRH antagonist cycle (p=0.065) (Table 2).

**Table 2:** Comparison of retrieved oocyte results (overall): univariate analysis The results of univariate analysis do not show a significant difference between the two groups, but for the number of blastocysts, the PPOS cycle group shows a trend (p=0.065). p-value: Student’s t-test. GnRH: gonadotropin-releasing hormone, PPOS: progesterone-primed ovarian stimulation

No. of embryo	GnRH antagonist cycle	PPOS cycle	p-value
	(n=149 cycles)	(n=147 cycles)	
No. of retrieved oocytes	8.9 ± 6.4	9.4 ± 5.6	0.465
No. of blastocysts	3.4 ± 3.2	4.1 ± 3.2	0.065

**Figure 2:**
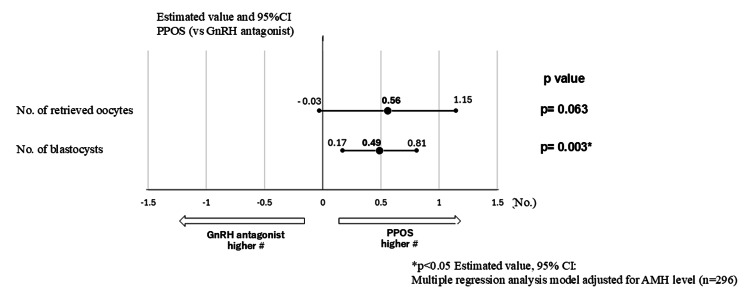
Comparison of retrieved oocyte results (overall): multivariate analysis In the background characteristics of the two groups, a trend (p<0.150) is observed for differences in total follitropin delta dosage, baseline FSH level, and AMH level. However, since the three factors show a strong correlation, only the AMH level is used as a moderating variable in the multivariate analysis. The results of estimates with a multiple regression analysis model show a significant trend for oocyte retrieval results with the PPOS cycle compared with the GnRH antagonist cycle (p=0.063), and a significantly higher number of blastocysts (+0.49 blastocysts, 95% CI: 0.17-0.51, p=0.003 vs. GnRH antagonist cycle). PPOS: progesterone-primed ovarian stimulation, GnRH: gonadotropin-releasing hormone, CI: confidence interval, FSH: follicle-stimulating hormone, AMH: anti-Müllerian hormone

In the background characteristics for both cycles, trends were observed in the differences in total dosage of follitropin delta and baseline FSH and AMH levels (p<0.150). However, since these three factors showed a strong correlation, the AMH level was used as a moderating variable in the multivariate analysis. In the multiple regression analysis model adjusted for AMH level, the PPOS cycle showed a trend toward a higher number of retrieved oocytes than the GnRH antagonist cycle (+0.56 oocytes, 95% confidence interval (CI): -0.03-1.15, p=0.063 vs. GnRH antagonist cycle). The number of blastocysts was also significantly higher (+0.49 blastocysts, 95% CI: 0.17-0.81, p=0.003 vs. GnRH antagonist cycle) (Figure 2). In addition, a stratified analysis by age (<35, 35-39, ≥40 years) was conducted (Table 3).

**Table 3:** Comparison of retrieved oocyte results by age group: univariate analysis No significant difference is seen between the two groups on univariate analysis in each age group. However, the number of blastocysts in the 35-39 age group is numerically higher in the PPOS group, and a trend is observed (p=0.063). p-value: Student’s t-test. GnRH: gonadotropin-releasing hormone, PPOS: progesterone-primed ovarian stimulation, G: GnRH antagonist cycle, P: PPOS cycle

Age group (y)	No. of retrieved oocytes or blastocysts	GnRH antagonist cycle	PPOS cycle	p-value
<35 y	No. of retrieved oocytes	10.9 ± 5.6	11.7 ± 6.3	0.534
(G: n=48, P: n=38)	No. of blastocysts	4.7 ± 5.4	5.4 ± 3.1	0.268
35-39 y	No. of retrieved oocytes	8.7 ± 7.4	9.1 ± 5.4	0.718
(G: n=66, P: n=70)	No. of blastocysts	3.2 ± 3.5	4.2 ± 3.3	0.083
≥40 y	No. of retrieved oocytes	6.4 ± 4.4	7.6 ± 4.7	0.255
(G: n=35, P: n=39)	No. of blastocysts	2.2 ± 0.4	2.7 ± 2.3	0.332

**Figure 3:**
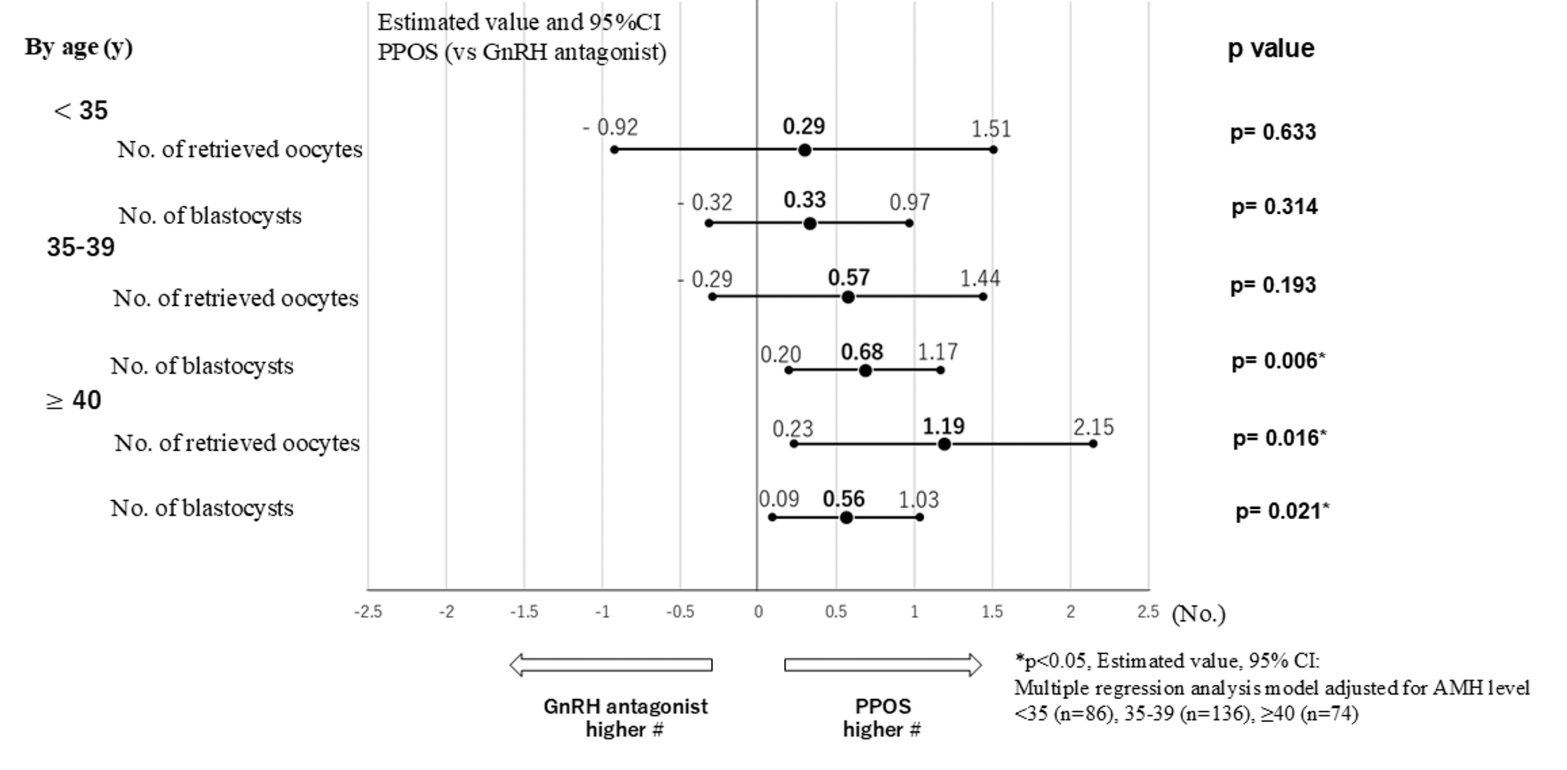
Comparison of retrieved oocyte results by age group: multivariate analysis The results of multiple regression analysis using AMH level as the moderating variable for each age group show that the number of blastocysts in the 35-39 years age group (+0.68 blastocysts, 95% CI: 0.20-1.17, p=0.006 vs. GnRH antagonist cycle), the number of retrieved oocytes (+1.19 oocytes, 95% CI: 0.23-2.15, p=0.016 vs. GnRH antagonist cycle), and the number of blastocysts (+0.56 blastocysts, 95% 95%CI: 0.09-1.03, p=0.021 vs. GnRH antagonist cycle) in the ≥40 years age group are significantly higher in the PPOS group. CI: confidence interval, PPOS: progesterone-primed ovarian stimulation, GnRH: gonadotropin-releasing hormone, AMH: anti-Müllerian hormone

The univariate analysis showed that the number of retrieved oocytes was 10.9 ± 5.6 versus 11.7 ± 6.3 (p=0.534) in the <35 years age group, 8.7 ± 7.4 versus 9.1 ± 5.4 (p=0.718) in the 35-39 years age group, and 6.4 ± 4.4 versus 7.6 ± 4.7 (p=0.255) in the ≥40 years age group. No significant differences were observed in any of the age groups. The number of blastocysts was 4.7 ± 5.4 versus 5.4 ± 3.1 (p=0.268) in the <35 years age group, 3.2 ± 3.5 versus 4.2 ± 3.3 (p=0.083) in the 35-39 years age group, and 2.2 ± 0.4 versus 2.7 ± 2.3 (p=0.332) in the ≥40 years age group. A trend toward a higher number of blastocysts with the PPOS cycle was observed in the 35-39 years age group (p=0.083). The results of the multiple regression analysis model, using the AMH level as the moderating variable in each age group, are described below (Figure 3).

In the <35 years age group, the number of retrieved oocytes was +0.29 (95% CI: -0.92-1.51, p=0.633 vs. the GnRH antagonist cycle), and the number of blastocysts was +0.33 (95% CI: -0.32-0.97, p=0.314 vs. the GnRH antagonist cycle). In the 35-39 years age group, the number of retrieved oocytes was +0.57 (95% CI: -0.29-1.44, p=0.193 vs. the GnRH antagonist cycle), and the number of blastocysts was +0.68 (95% CI: 0.20-1.17, p=0.006 vs. the GnRH antagonist cycle). In the ≥40 years age group, the number of retrieved oocytes was +1.19 (95% CI: 0.23-2.15, p=0.016 vs. the GnRH antagonist cycle), and the number of blastocysts was +0.56 (95% CI: 0.09-1.03, p=0.021 vs. the GnRH antagonist cycle). The number of blastocysts in the 35-39 years age group (p=0.006), as well as both the number of oocytes (p=0.016) and the number of blastocysts (p=0.021) in the ≥40 years age group, were significantly higher with the PPOS cycle compared to the GnRH antagonist cycle.

Pregnancy outcomes are described below. Clinical pregnancy was defined as a gestational sac confirmed in the uterus by six weeks of gestation, and ongoing pregnancy was defined as confirmation of a fetal heartbeat at 10 weeks of gestation. In the GnRH antagonist cycle versus the PPOS cycle, the clinical pregnancy rate was 66.7% versus 63.2%, and the ongoing pregnancy rate was 66.7% versus 55.2%. On univariate analysis (Table 4), the clinical pregnancy rate was 66.7% versus 63.2% (p=0.750), and the ongoing pregnancy rate was 66.7% versus 55.2% (p=0.160). No significant difference was observed between the two cycles in either the clinical pregnancy rate or the ongoing pregnancy rate.

**Table 4:** Pregnancy outcome per transfer (overall): univariate analysis The results of univariate analysis do not show a significant difference between the groups for either the clinical pregnancy rate or the ongoing pregnancy rate. p-value: Fisher’s exact test. GnRH: gonadotropin-releasing hormone, PPOS: progesterone-primed ovarian stimulation

Pregnancy rate	GnRH antagonist cycle	PPOS cycle	p-value
	(n=72 cycle)	(n=106 cycle)	
Clinical pregnancy rate	48 (66.7%)	67 (63.2%)	0.75
Ongoing pregnancy rate	48 (66.7%)	58 (55.2%)	0.16

**Figure 4:**
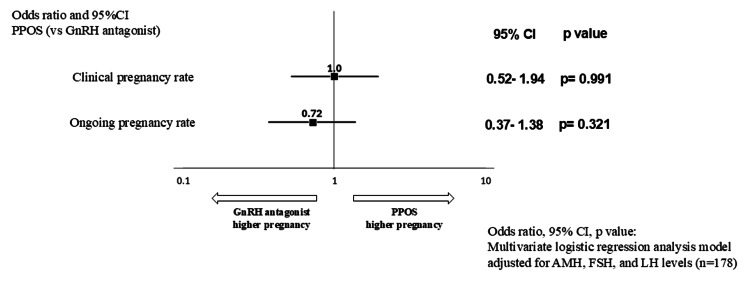
Pregnancy outcome per transfer (overall): multivariate analysis In the background of patients who underwent transfer during the transfer cycle, a trend is observed for differences in AMH, FSH, and LH levels between the groups. Therefore, a multivariate logistic regression analysis was performed using these three factors as moderating variables. The odds ratio and confidence interval were estimated; however, a significant difference between the groups, similar to that observed in the univariate analysis, was not found. CI: confidence interval, PPOS: progesterone-primed ovarian stimulation, GnRH: gonadotropin-releasing hormone, AMH: anti-Müllerian hormone, FSH: follicle-stimulating hormone, LH: luteinizing hormone

Among patients who underwent embryo transfers in the transfer cycle, a trend was observed in the differences in AMH, FSH, and LH levels between the PPOS and GnRH antagonist cycles. Therefore, a multivariate logistic regression analysis was conducted using these three factors as moderating variables. The odds ratios and confidence intervals were estimated (Figure 4).

Similar to the univariate analysis, the results showed no significant differences between the two cycles in terms of clinical pregnancy rate (+1.0, 95% CI: 0.52-1.94, p=0.991 vs. GnRH antagonist cycle) or ongoing pregnancy rate (+0.72, 95% CI: 0.37-1.38, p=0.321 vs. GnRH antagonist cycle). In addition, a stratified analysis by age (<35, 35-39, ≥40 years) was conducted for the same variables (Table 5).

**Table 5:** Pregnancy outcome per transfer by age group: univariate analysis The results of univariate analysis by age group do not show a significant difference between the groups in either the clinical pregnancy rate or the ongoing pregnancy rate. p-value: Fisher’s exact test. GnRH: gonadotropin-releasing hormone, PPOS: progesterone-primed ovarian stimulation, G: GnRH antagonist cycle, P: PPOS cycle

Age group (y)	Pregnancy rate	GnRH antagonist cycle	PPOS cycle	p-value
<35	Clinical pregnancy rate	27 (84.4%)	25 (73.5%)	0.371
(G: n=32, P: n=34)	Ongoing pregnancy rate	27 (84.4%)	23 (67.7%)	0.154
35-39	Clinical pregnancy rate	16 (53.3%)	34 (65.4%)	0.349
(G: n=30, P: n=52)	Ongoing pregnancy rate	16 (53.3%)	29 (56.9%)	0.819
≥40	Clinical pregnancy rate	5 (50.0%)	10 (50.0%)	0.706
(G: n=10, P: n=20)	Ongoing pregnancy rate	4 (40.0%)	6 (30.0%)	0.425

**Figure 5:**
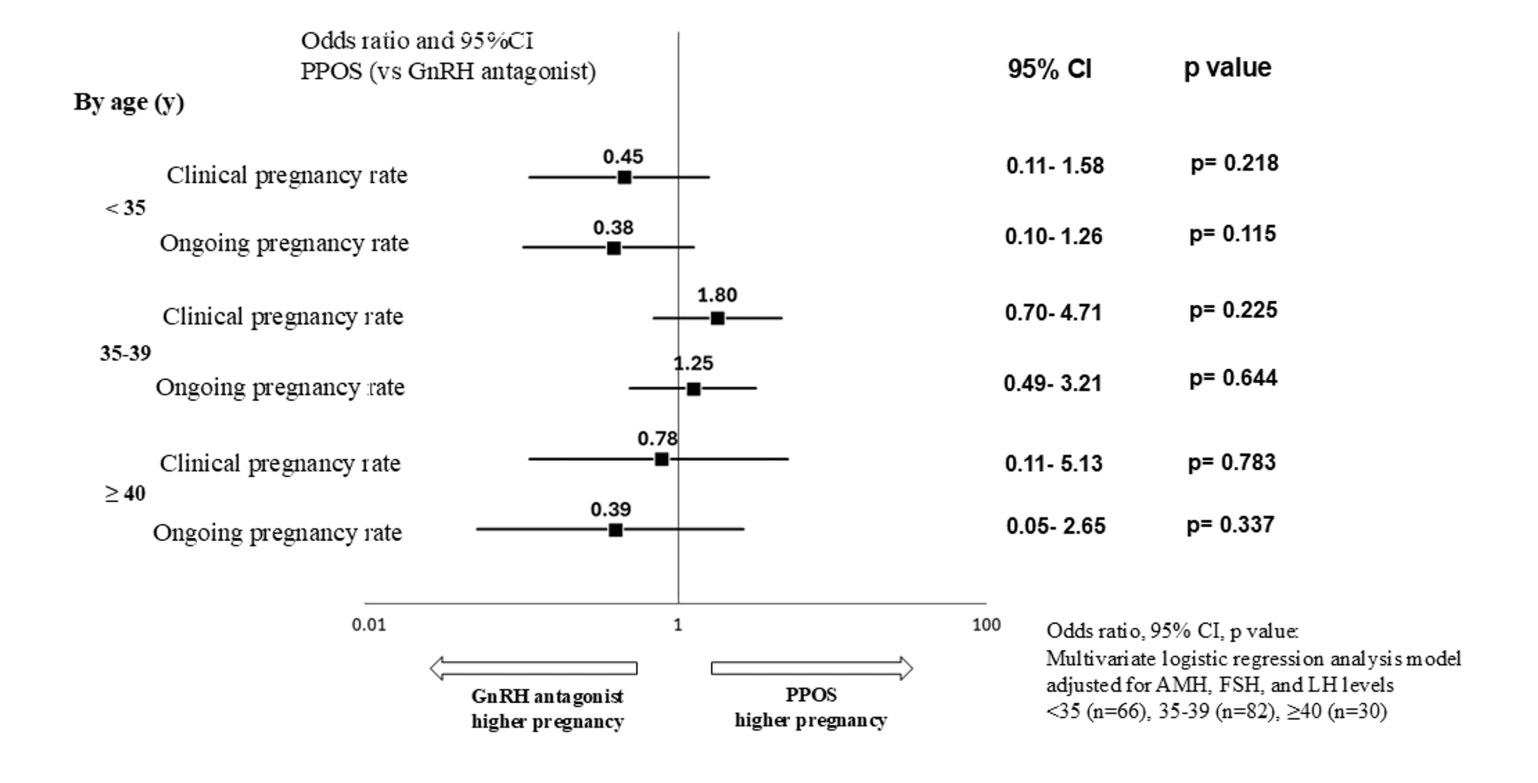
Pregnancy outcome per transfer by age group: multivariate analysis A multivariate logistic regression analysis using AMH, FSH, and LH levels as moderating variables was conducted for each age group, and the odds ratios and confidence intervals were estimated. However, significant differences similar to those in the univariate analysis are not seen between the two protocols in any age group. CI: confidence interval, PPOS: progesterone-primed ovarian stimulation, GnRH: gonadotropin-releasing hormone, AMH: anti-Müllerian hormone, FSH: follicle-stimulating hormone, LH: luteinizing hormone

The clinical pregnancy rate with the GnRH antagonist cycle versus the PPOS cycle was 84.4% versus 73.5% (p=0.371) in the <35 years age group, 53.3% versus 65.4% (p=0.349) in the 35-39 years age group, and 50.0% versus 50.0% (p=0.706) in the ≥40 years age group. No significant differences were observed in any of the age groups. The ongoing pregnancy rate was 84.4% versus 67.7% (p=0.154) in the <35 years age group, 53.3% versus 56.9% (p=0.819) in the 35-39 years age group, and 40.0% versus 30.0% (p=0.425) in the ≥40 years age group. The results of the univariate analysis for each age group showed no significant differences between the two cycles in either the clinical pregnancy rate or the ongoing pregnancy rate. Multivariate logistic regression analyses were conducted using AMH, FSH, and LH levels as moderating variables in each age group, and odds ratios and confidence intervals were estimated. However, similar to the univariate analysis, no significant differences were observed between the two cycles in any of the age groups, as shown below (Figure 5).

In the <35 years age group, the clinical pregnancy rate was +0.45 (95% CI: 0.11-1.58, p=0.218 vs. the GnRH antagonist cycle), and the ongoing pregnancy rate was +0.38 (95% CI: -0.10-1.26, p=0.115 vs. the GnRH antagonist cycle). In the 35-39 years age group, the clinical pregnancy rate was +1.80 (95% CI: 0.70-4.71, p=0.225 vs. the GnRH antagonist cycle), and the ongoing pregnancy rate was +1.25 (95% CI: 0.49-3.21, p=0.644 vs. the GnRH antagonist cycle). In the ≥40 years age group, the clinical pregnancy rate was +0.78 (95% CI: 0.11-5.13, p=0.783 vs. the GnRH antagonist cycle), and the ongoing pregnancy rate was +0.39 (95% CI: 0.05-2.65, p=0.337 vs. the GnRH antagonist cycle).

## Discussion

In this study, the ovarian response to stimulation with follitropin delta was compared between two protocols: the GnRH antagonist protocol, a conventional method of ovarian stimulation, and the PPOS protocol, a relatively new approach. The purpose was to examine the effects on oocyte retrieval, culture outcomes, and pregnancy rates in Japanese women undergoing ovarian stimulation for ART. In terms of the number of oocytes retrieved and blastocysts formed, the PPOS cycle tended to yield more oocytes and showed a significantly higher number of blastocysts. Across all age groups, the PPOS cycle generally resulted in higher numbers of retrieved oocytes and blastocysts. However, no significant difference was observed in the pregnancy rate per transfer. Reports on the PPOS protocol using follitropin delta remain limited worldwide, and this study provides the first evidence for its use in Japanese (Asian) women, including analysis up to the pregnancy stage.

Ovarian stimulation in this study was performed using follitropin delta, comparing the GnRH antagonist and PPOS protocols under a freeze-all policy. The PPOS protocol is a relatively new COS method, first reported by Kuang et al. [6]. It has been increasingly adopted in Japan in recent years due to its simplicity, ease of use, and reduced financial burden on patients [7]. Although some studies have compared the GnRH antagonist protocol, currently widely used in COS, with the PPOS protocol [10], reports specifically involving follitropin delta are scarce. This study demonstrated that, with follitropin delta, the PPOS cycle tends to result in a greater number of retrieved oocytes and blastocysts. However, no significant difference was found in pregnancy rate per transfer. These results are consistent with previous studies using other medications.

This may be attributed to the PPOS protocol’s simplified ovulation control and the flexibility in timing oocyte retrieval, as it assumes frozen embryo transfer. Given this context, it may represent a turning point in which clinician management yields a higher number of oocytes and blastocysts compared to the GnRH antagonist cycle. Furthermore, since frozen embryo transfer accounts for over 90% of births resulting from in vitro fertilization in Japan, the inability to perform fresh embryo transfer in the PPOS protocol is not considered a major drawback [20]. Although obtaining more oocytes and blastocysts might suggest improved outcomes, this study, like previous ones using other drugs, found no difference in pregnancy results [11]. If further studies confirm these findings, it may prompt a re-evaluation of the necessity of striving for higher numbers of oocytes and blastocysts.

In previous studies, including clinical trials (ESTHER-1 [12], STORK [13], GRAPE [14]), the majority of reports on follitropin delta involved fresh embryo transfer. Reports on frozen embryo transfer are limited. All systematic reviews and meta-analyses on follitropin delta published to date have focused on fresh embryo transfer [15-17]. Evidence regarding frozen embryo transfer, which is becoming increasingly common worldwide, is limited [18].

A study similar to the present one, in which ovarian stimulation was performed using follitropin delta and PPOS and GnRH antagonist protocols with dydrogesterone, was reported from Brazil in 2025 [21]. The objective of that study was to evaluate oocyte retrieval performance, and the results concluded that there was no significant difference between the two protocols. A similar study has also been reported from Japan, and consistent with the present findings, the number of retrieved oocytes and blastocysts was higher with the PPOS protocol [22].

A summary of some of the reports on follitropin delta to date is provided below. In a Romanian study from 2021, the number of retrieved oocytes was 10.48 ± 6.57 (mean ± SD) for women under 35 years and 7.65 ± 5.50 for women over 35 years [23]. In a French study from 2023, the number of retrieved oocytes was 11.3 ± 6.8, and the ongoing pregnancy rate was 29.6% [24]. In a multinational observational study from 2022, the median (interquartile range) number of retrieved oocytes was 5.0 (2.0, 7.0) for AMH <7 pmol/L, 8.0 (6.0, 12.0) for AMH 7 to <15 pmol/L, 12.0 (9.0, 17.0) for AMH 15 to <35 pmol/L, and 12.0 (7.0, 18.0) for AMH >35 pmol/L. In an analysis by age group, the number of retrieved oocytes was 11.8 ± 7.64 for women under 35 years and 5.3 ± 4.66 for those aged 40 years and older. The ongoing pregnancy rate was 27.0% [25]. In a 2025 German study analyzing registry data, the number of retrieved oocytes was 11.0 ± 7.2, and the pregnancy rate was 38.0% [26].

Thus, the results of the present study are consistent with previously reported studies regarding the number of retrieved oocytes, and they also align with reported findings in women over 40 years of age. While results involving fresh embryo transfer have been reported in several other countries, most reports from Japan pertain to frozen embryo transfer. A summary of some of these reports follows.

In a 2024 multicenter joint study from Japan, in which our hospital participated, the number of retrieved oocytes using follitropin delta for ovarian stimulation was 11.0 (range: 0.00-44.00), and the live birth rate at the first embryo transfer was 51.5%. Regarding cumulative pregnancy and live birth rates, cost-effectiveness was significantly higher with follitropin delta compared to the control group, suggesting its effectiveness [27].

For PPOS protocols, a 2024 report from Japan compared fixed and flexible PPOS protocols [7]. The number of retrieved oocytes was 12.13 ± 6.74 with fixed PPOS versus 10.77 ± 6.22 with flexible PPOS (p=0.240), and the ongoing pregnancy rate was 43.4% with fixed PPOS versus 54.5% with flexible PPOS (p=0.526). These results are comparable to those of the present study (PPOS cycle: retrieved oocytes 9.4 ± 5.6, ongoing pregnancy rate 55.2%).

Follitropin delta is a recombinant FSH preparation, with its daily dosage determined by AMH level and body weight. It employs an individualized dosing algorithm in which the dosage remains fixed throughout the stimulation period. As the first recombinant FSH preparation derived from human cells, follitropin delta is expected to act more physiologically in vivo. Moving forward, it is anticipated that its use will become more widespread in patients with diminished ovarian function.

The authors have conducted investigations comparing conventional HMG preparations with follitropin delta [28] and flexible versus fixed protocols during oocyte stimulation using a GnRH antagonist protocol with follitropin delta [29]. Additionally, they have examined the initiation of the GnRH antagonist on day 5 versus day 6 within a fixed protocol [19]. All of these assessments were conducted using freeze-all protocols.

We hypothesized that the pregnancy rates with the GnRH antagonist protocol and the PPOS protocol would be equivalent. This study was conducted to compare the protocols and verify the hypothesis. Compared with reports on ovarian stimulation using follitropin delta worldwide to date, the present results showed a roughly equal number of retrieved oocytes and an equal or higher pregnancy rate. With the standardization of treatment using follitropin delta, it has become easier to compare efficacy and safety between institutions globally. The PPOS protocol in the present study has the advantage of reducing the number of clinic visits for patients. The starting dosage of follitropin delta can be determined based on AMH level and body weight, and the dose does not need to be changed during ovarian stimulation. Combining these factors may enable the standardization of ART treatment methods for doctors, ranging from those with limited experience to those with extensive experience. This will provide new evidence for ART, a field where various treatment methods have been combined, and is promising for the accumulation of consistent data worldwide in the future.

A limitation of this study is that, since the use of preimplantation genetic testing is not covered by national health insurance in Japan, embryo quality in this study was assessed using time-lapse imaging, as previously described, and evaluated based on the Gardner classification. Regarding embryo quality, a report comparing follitropin delta with follitropin alpha and follitropin beta demonstrates no difference [30]. However, issues such as differences in euploid embryos resulting from the two stimulation methods will need to be investigated in the future. Additionally, this study was retrospective and nonrandomized, which means potential treatment duration and selection biases are possible, and final live birth rates were not obtained.

## Conclusions

This research offers important insights into the comparative effectiveness of GnRH antagonist and PPOS protocols using follitropin delta in Japanese women undergoing ART. By analyzing a substantial dataset from a single IVF clinic over a two-year period, the study demonstrates that while pregnancy rates were comparable between the two protocols, the PPOS protocol showed a trend toward improved oocyte and blastocyst outcomes across all age groups. Large-scale prospective studies will be needed in the future.
